# A Flexible Electrochemical Sensor Based on Porous Ceria Hollow Microspheres Nanozyme for Sensitive Detection of H_2_O_2_

**DOI:** 10.3390/bios15100664

**Published:** 2025-10-02

**Authors:** Jie Huang, Xuanda He, Shuang Zou, Keying Ling, Hongying Zhu, Qijia Jiang, Yuxuan Zhang, Zijian Feng, Penghui Wang, Xiaofei Duan, Haiyang Liao, Zheng Yuan, Yiwu Liu, Jinghua Tan

**Affiliations:** 1 School of Packaging Engineering, Hunan University of Technology, Zhuzhou 412007, China; huangjie3@alumni.sjtu.edu.cn (J.H.); m24085600021@stu.hut.edu.cn (X.H.); 19374285193@163.com (S.Z.); 13768127226@163.com (K.L.); 15823262606@163.com (H.Z.); 17872584038@163.com (Q.J.); fzj6900@163.com (Z.F.); dxf0923@163.com (X.D.); haiyangliao1990@163.com (H.L.); liuyiwu@hut.edu.cn (Y.L.); 2 School of Biomedical Engineering, Guangzhou Medical University, Guangzhou 511436, China; 3 Hunan Provincial Key Laboratory of Environmental Catalysis & Waste Recycling, Hunan Institute of Engineering, College of Materials and Chemical Engineering, Xiangtan 411104, China; yuxuanzhang1990@163.com; 4 State Key Laboratory of Metal Matrix Composites, School of Chemistry and Chemical Engineering, Shanghai Jiao Tong University, Shanghai 200240, China; phwang0622@sjtu.edu.cn; 5 Institute of Chinese Materia Medica, China Academy of Chinese Medical Sciences, Beijing 100022, China

**Keywords:** ceria, porous hollow microspheres, nanoenzyme, screen-printed electrode, H_2_O_2_ biosensor

## Abstract

The development of cost-effective and highly sensitive hydrogen peroxide (H_2_O_2_) biosensors with robust stability is critical due to the pivotal role of H_2_O_2_ in biological processes and its broad utility across various applications. In this work, porous ceria hollow microspheres (CeO_2_-phm) were synthesized using a solvothermal synthesis method and employed in the construction of an electrochemical biosensor for H_2_O_2_ detection. The resulting CeO_2_-phm featured a uniform pore size centered at 3.4 nm and a high specific surface area of 168.6 m^2^/g. These structural attributes contribute to an increased number of active catalytic sites and promote efficient electrolyte penetration and charge transport, thereby enhancing its electrochemical sensing performance. When integrated into screen-printed carbon electrodes (CeO_2_-phm/cMWCNTs/SPCE), the CeO_2_-phm/cMWCNTs/SPCE-based biosensor exhibited a wide linear detection range from 0.5 to 450 μM, a low detection limit of 0.017 μM, and a high sensitivity of 2070.9 and 2161.6 μA·mM^−1^·cm^−2^—surpassing the performance of many previously reported H_2_O_2_ sensors. In addition, the CeO_2_-phm/cMWCNTs/SPCE-based biosensor possesses excellent anti-interference performance, repeatability, reproducibility, and stability. Its effectiveness was further validated through successful application in real sample analysis. Hence, CeO_2_-phm with solvothermal synthesis has great potential applications as a sensing material for the quantitative determination of H_2_O_2_.

## 1. Introduction

Hydrogen peroxide (H_2_O_2_), a representative reactive oxygen species (ROS), plays a pivotal role in various physiological and pathological processes, including immune defense, redox signaling, and oxidative stress regulation [1,2]. However, abnormal accumulation of H_2_O_2_ is closely associated with the onset and progression of numerous diseases such as cancer, cardiovascular dysfunctions, and neurodegenerative disorders [3,4]. Beyond its biological relevance, H_2_O_2_ is also widely employed as a bleaching, sterilizing, and oxidizing agent in industrial production, food processing, and agriculture [5,6]. Residual or excessive H_2_O_2_ in the environment-especially in wastewater and food systems-can induce oxidative damage in ecosystems and raise serious health concerns [7,8]. Therefore, the development of sensitive, reliable, and cost-effective strategies for H_2_O_2_ detection is of great significance for biomedical diagnostics, environmental surveillance, and food safety monitoring [9].

To date, various analytical methods have been developed for the detection of H_2_O_2_, including titration, chromatography, fluorescence, colorimetry, and electrochemical sensing [10,11]. Among these, titration and chromatography often involve complicated operations and large sample consumption, while fluorescence and colorimetric assays are prone to signal interference, photobleaching, and limited quantitative precision [10]. In contrast, electrochemical methods have gained increasing attention due to their simplicity, low cost, rapid response, high sensitivity, and potential for miniaturization and in-field detection [12]. In electrochemical sensors, the performance of the working electrode critically determines the sensitivity and selectivity of the detection process. Although a variety of enzyme-based electrodes have been proposed for H_2_O_2_ detection due to their excellent catalytic efficiency, they suffer from intrinsic drawbacks such as high cost, poor long-term stability, and strict environmental requirements (e.g., pH and temperature), which hinder their practical applications [13].

To address these limitations, researchers have turned to nanozymes—engineered nanomaterials that mimic the catalytic function of natural enzymes [14,15]. Compared with their biological counterparts, nanozymes exhibit several advantages, including higher stability under harsh conditions, ease of mass production, lower cost, and tunable surface properties [16]. These features make nanozyme-based electrochemical sensors a promising alternative to enzyme-based systems for H_2_O_2_ detection. Among the various nanozyme candidates, cerium dioxide (CeO_2_) has emerged as a particularly attractive material due to its excellent redox reversibility (Ce^3+^/Ce^4+^), strong ROS scavenging ability, and good biocompatibility [17]. These properties enable CeO_2_ to effectively catalyze redox reactions involving H_2_O_2_ [18].

However, conventional CeO_2_ nanostructures such as nanoparticles, nanorods, and nanocubes often exhibit low specific surface areas and limited accessibility of catalytic sites, which restrict their electrocatalytic efficiency [19,20,21]. In addition, poor mass and electron transport within dense or aggregated structures further hinders sensor performance. To overcome these issues, constructing CeO_2_ with rationally engineered hierarchical nanostructures has become a focus of recent research. The hollow mesoporous structure offers a particularly attractive solution, including high surface area, enhanced active site exposure, efficient diffusion channels, and favorable electron transfer kinetics. The hollow interior acts as a confined reaction chamber for signal amplification, while the mesoporous shell allows for rapid electrolyte infiltration and mass transport [22,23]. These synergistic features are highly desirable for boosting the electrochemical sensing performance of non-enzymatic H_2_O_2_ sensors.

In parallel with the advancement of sensing materials, the development of flexible electrochemical sensors has also attracted growing interest due to their mechanical adaptability, light weight, and potential for wearable or on-site diagnostic applications. Flexible sensors, often based on screen-printed or polymer-supported electrodes, can conform to curved or dynamic surfaces such as skin, textiles, or food packaging, offering unique advantages in real-time, non-invasive, and point-of-care monitoring [24,25,26]. When integrated with nanozyme materials, flexible platforms can further enhance sensor accessibility and application scope while maintaining high electrochemical performance [27].

**Scheme 1:**
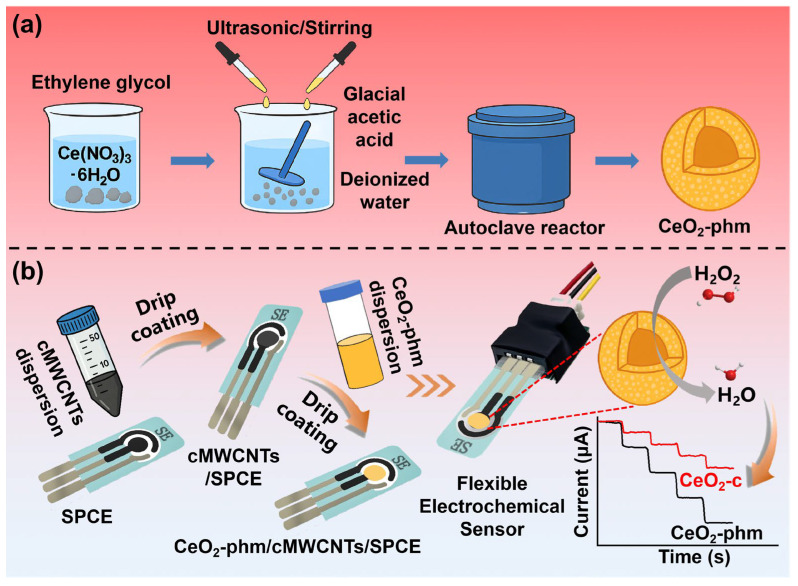
Schematics of the preparation of CeO_2_-phm (**a**) and schematic for fabrication of the H_2_O_2_ biosensor and electrochemical detection of H_2_O_2_ (**b**).

In this work, we report the application of porous ceria hollow microspheres (CeO_2_-phm) as an advanced nanozyme material for the construction of a high-performance flexible, non-enzymatic electrochemical sensor for H_2_O_2_ detection. As illustrated in Scheme 1, CeO_2_-phm was prepared through a simple solvothermal method and subsequently incorporated onto a screen-printed carbon electrode (SPCE) pre-functionalized with carboxylated multi-walled carbon nanotubes (cMWCNTs), yielding the CeO_2_-phm/cMWCNTs/SPCE sensor. The fabricated biosensor displayed remarkable electrocatalytic performance for the reduction of H_2_O_2_, characterized by a wide linear response range, an ultralow detection limit, and a high sensitivity. Compared with electrochemical sensors based on commercial CeO_2_ nanospheres with solid cores (CeO_2_-c), it demonstrates significantly higher sensitivity. In addition, it exhibited excellent selectivity, repeatability, reproducibility and stability. Its practical applicability was validated by the successful quantification of H_2_O_2_ in real sample matrices. To the best of our knowledge, although CeO_2_-phm materials have been extensively investigated in catalytic and biomedical applications, their integration with flexible sensing platforms for electrochemical H_2_O_2_ detection has not yet been reported. This work not only expands the functional scope of CeO_2_-phm in biosensing but also offers a promising strategy for the development of flexible, low-cost, and high-performance nanozyme-based sensors for real-world applications in healthcare, environmental monitoring, and food safety.

## 2. Materials and Methods

### 2.1. Materials and Reagents

Cerium nitrate hexahydrate (Ce(NO_3_)_3_·6H_2_O), ethylene glycol (C_2_H_6_O_2_, EG), glacial acetic acid (CH_3_COOH), ethanol, ascorbic acid (AA), glucose (GLu), citric acid (CA), uric acid (UA) and sodium chloride (NaCl) were purchased from Sigma-Aldrich. Phosphate-buffered saline (PBS, pH 7.0) was prepared using standard protocol. All chemicals were analytical grade and used without further purification. Deionized water (resistivity ≥ 18.2 MΩ·cm) was used throughout the experiments. Carboxyl multi-walled carbon nanotube (cMWCNTs) and commercial CeO_2_ nanospheres with solid cores (CeO_2_-c) were purchased from Nanjing XFNANO Materials Tech Co., Ltd. Screen-printed carbon electrodes (SPCE) were purchased from Poten Technology Co., Ltd (Weihai, China). Fetal Bovine Serum (FBS) was obtained from Thermo Fisher Scientific (Carlsbad, CA, USA).

### 2.2. Synthesis of CeO_2_-phm

CeO_2_-phm was synthesized via a one-pot solvothermal method using cerium nitrate as the cerium source and ethylene glycol as both solvent and mild reducing agent. In a typical synthesis, 2.0 g of cerium nitrate hexahydrate (Ce(NO_3_)_3_·6H_2_O) was dissolved in 80 mL of ethylene glycol under ultrasonic agitation to ensure complete dissolution. Subsequently, 4 mL of deionized water and 4 mL of glacial acetic acid were added to the solution. The mixture was vigorously stirred for 30 min to form a homogeneous precursor solution. The resulting solution was then transferred into a Teflon-lined stainless-steel autoclave and maintained at 180 °C for 6 h under static conditions. Upon natural cooling to ambient temperature, the resulting yellow precipitate was isolated by centrifugation. To eliminate residual inorganic ions and organic impurities, the collected solid was repeatedly washed with deionized water and ethanol. The purified material was then dried in an oven at 80 °C overnight, affording the final CeO_2_-phm powder.

### 2.3. Characterization

The morphology and microstructure of CeO_2_-phm were investigated by field-emission scanning electron microscopy (FE-SEM, JEOL JSM-7500F) and transmission electron microscopy (TEM, JEOL JEM-2100). Elemental distribution and compositional information were obtained through energy-dispersive X-ray spectroscopy (EDS) integrated with the SEM. The crystalline phase was identified using X-ray diffraction (XRD, Rigaku SmartLab) with Cu Kα radiation (λ = 1.5406 Å), scanning over a 2θ range of 10–90° at a rate of 5°·min^−1^. Textural characteristics, including specific surface area and pore size distribution, were analyzed by nitrogen adsorption–desorption isotherms using a Micromeritics ASAP 2460 system and evaluated via the Brunauer–Emmett–Teller (BET) method. Surface elemental states and chemical composition were characterized by X-ray photoelectron spectroscopy (XPS, Thermo Scientific ESCALAB 250Xi, Thermo Fisher Scientific, Inc., Waltham, MA, USA). Optical absorption properties were recorded through UV–Vis diffuse reflectance spectroscopy (DRS) on a Shimadzu UV-2600 equipped with an integrating sphere. Furthermore, particle dispersion behavior and colloidal stability were assessed via dynamic light scattering (DLS) and zeta potential analysis using a Malvern Zetasizer Nano ZS90 (Malvern Panalytical Ltd., Malvern, UK). Prior to measurement, the powder samples were ultrasonically dispersed in deionized water at a concentration of 0.1 mg·mL^−1^.

### 2.4. Fabrication of the Flexible Electrochemical Biosensor

Screen-printed carbon electrodes (SPCE) on flexible PET substrates were used as the biosensor base. The 10 μL cMWCNTs suspension (1 mg/mL) was dropped on the working electrode area and allowed to dry under ambient conditions. The obtained electrode was denoted as cMWCNTs/SPCE. Next, the working electrode was immersed in an aqueous solution containing 1 mg·mL^−1^ EDC and NHS for 30 min to activate the carboxyl functional groups on the MWCNT surface. Following activation, the electrode was thoroughly rinsed with ultrapure water to eliminate unreacted coupling agents. Then, a dispersion of 1 mg/mL CeO_2_-phm in ethanol was drop-cast (6 μL) onto the working electrode area and allowed to dry under ambient conditions. The modified electrodes were gently rinsed with water to remove loosely bound particles. The resulting CeO_2_-phm/cMWCNTs/SPCE was stored at room temperature until use. Replacing CeO_2_-c with CeO_2_-phm can obtain CeO_2_-c/cMWCNTs/SPCE.

### 2.5. Electrochemical Measurements

Electrochemical performance was evaluated using a CHI660E electrochemical workstation (CH Instruments, shanghai, China) in a conventional three-electrode setup. Amperometric measurements were performed in 10 mL of 0.1 M PBS (pH 7.0) at a constant potential of −0.55 V versus Ag/AgCl under magnetic stirring. H_2_O_2_ was sequentially added to the solution to assess biosensor response. Selectivity tests were carried out by adding potential interferents (ascorbic acid, glucose, citric acid, uric acid, sodium chloride) at physiologically relevant concentrations. For real sample analysis, groundwater, commercial drinking water, milk and fetal bovine serum were spiked with known amounts of H_2_O_2_ and tested under identical conditions.

## 3. Results

### 3.1. Preparation and Characterization of CeO_2_-phm

Scheme 1a displays the synthetic route of CeO_2_-phm. CeO_2_-phm was synthesized through a one-pot solvothermal reaction, in which cerium nitrate served as the cerium precursor, ethylene glycol acted simultaneously as the solvent and a mild reducing agent, while acetic acid and water functioned as structure-directing and hydrolysis-promoting agents, respectively.

**Figure 1:**
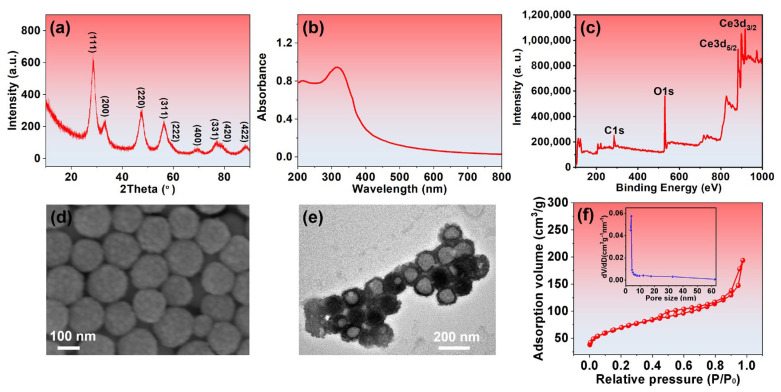
(**a**) XRD patterns of CeO_2_-phm; (**b**) UV-Visible absorbance spectrum of CeO_2_-phm; (**c**) XPS analysis of CeO_2_-phm; (**d**) SEM image of CeO_2_-phm; (**e**) TEM image of CeO_2_-phm; (**f**) N_2_ sorption isotherms and pore size distributions (inset) for CeO_2_-phm.

The X-ray diffraction (XRD) patterns for CeO_2_-phm revealed nine diffraction peaks (Figure 1a), which match well with the (111), (200), (220), (311), (222), (400), (331), (420) and (422) planes of cubic fluorite CeO_2_ (JCPDS No. 34-0394) [28]. In the FTIR spectrum of CeO_2_-phm (Figure S1), the absorption band at 3381 cm^−1^ is attributed to the stretching vibration of Ce–OH groups, while the peak at 1600 cm^−1^ corresponds to the stretching vibrations of Ce–O–C and C–O bonds [29]. The UV–Vis spectra of the CeO_2_-phm were shown in Figure 1b. The CeO_2_-phm exhibits a prominent absorption peak at 350 nm, which is attributed to the charge transfer transition from the O^2−^ 2p valence band to the Ce^4+^ 4f conduction band in CeO_2_ [30]. X-ray photoelectron spectroscopy (XPS) characterization of CeO_2_-phm confirmed the existence of cerium species (Figure 1c). The characteristic peaks observed at 900.7, 882.3, 907.0, 888.6, 916.6, and 898.3 eV (denoted as u, v, u″, v″, u‴, and v‴, respectively) were assigned to Ce^4+^ species, while the additional signals at 902.9 eV (u′) and 885.3 eV (v′) corresponded to Ce^3+^ (Figure S2). Based on the XPS deconvolution analysis, the ratio of Ce^3+^/Ce^4+^ reaches 48.4%. The enzyme-mimetic activity of ceria is closely associated with the Ce^3+^/Ce^4+^ ratio. The elevated Ce^3+^ content in CeO_2_ correlates with a higher density of oxygen vacancies, as each pair of Ce^3+^ ions compensates for one oxygen vacancy [31,32]. In addition, the calculated atomic ratio of O and Ce by XPS is about 4.7, which is 2.35 times higher than that (2) in CeO_2_, also suggesting that CeO_2_-phm possesses a large amount of oxygen vacancies [22]. The presence of abundant oxygen vacancies facilitates oxygen adsorption and promotes rapid redox reactions, both of which are critical for enhancing the sensitivity and response time of CeO_2_-based electrochemical biosensors. These results confirm the successful synthesis of CeO_2_.

The hollow mesoporous structure of CeO_2_-phm was subsequently elucidated by electron microscopy and surface area analysis. SEM images (Figure 1d) revealed that the as-synthesized CeO_2_-phm exhibited uniform spherical morphology with average diameters of ~140 nm. The surface texture appears rough, indicating the aggregation of primary nanoparticles on the sphere surfaces, which suggests the formation of a porous shell. The closely packed structure with distinguishable interparticle boundaries is indicative of well-assembled nanocrystals constituting the microsphere shells. To further investigate the elemental distribution and compositional homogeneity, energy-dispersive X-ray spectroscopy (EDS) elemental mapping was performed. The EDS layered image (Figure S3a) of CeO_2_-phm shows the overall elemental distribution. Elemental mapping images for (Figure S3b) Ce, (Figure S3c) O, (Figure S3d) N, and (Figure S3e) C illustrate a uniform distribution of these elements throughout the sample. The corresponding EDS spectrum of CeO_2_-phm (Figure S3f), with the inset presenting the quantified elemental composition, confirms that Ce and O are the major constituents. As shown in Figure 1e, the individual particles exhibit a well-defined spherical morphology with a darker periphery and a relatively brighter center, indicating the presence of a hollow interior structure. Moreover, the shell of each microsphere appears to be composed of aggregated nanoparticles, forming a porous network. Nitrogen adsorption–desorption isotherms (Figure 1f) of CeO_2_-phm exhibited typical type IV behavior with an H3-type hysteresis loop, confirming the mesoporous nature of the sample [33,34]. The BET specific surface area up to 168.6 m^2^/g, and the Barrett–Joyner–Halenda (BJH) pore size distribution (inset of Figure 1f) showed a narrow peak centered at 3.4 nm. Such a porous structure not only increases the number of electroactive sites but also facilitates ion diffusion in sensing applications.

The hydrodynamic size and colloidal stability of CeO_2_-phm were assessed using dynamic light scattering (DLS) (Figure S4) and zeta potential measurements. DLS results revealed an average hydrodynamic diameter of approximately 152.2 nm with a low polydispersity index (PDI) of 0.154, indicating uniform particle distribution and good dispersion stability in aqueous media. The observed particle size from DLS was larger than that obtained by TEM, which can be attributed to the fact that DLS evaluates particles in their hydrated and dynamic state, while TEM measures the dried and immobilized counterparts under vacuum conditions [35]. The zeta potential measurement revealed a surface charge of −20.6 mV, confirming good electrostatic repulsion among particles and good colloidal stability [36], which is particularly beneficial for preparing modified electrodes through the droplet coating method.

### 3.2. Electrochemical Characterization of Modified Electrodes

**Figure 2:**
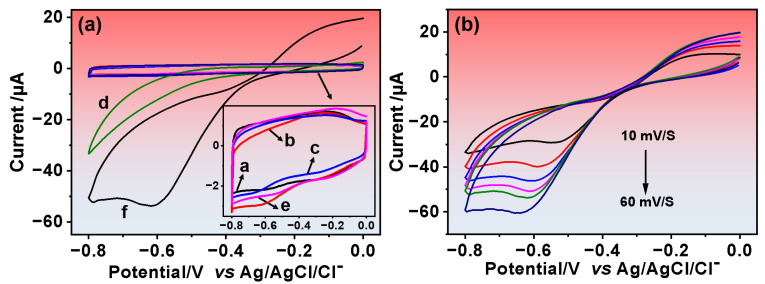
(**a**) Cyclic voltammograms (CVs) of cMWCNTs/SPCE, CeO_2_-c/cMWCNTs/SPCE and CeO_2_-phm/cMWCNTs/SPCE in 0.1 M PBS (pH 7.0) containing 50 μM H_2_O_2_ (a, c and e) or without H_2_O_2_ (b, d and f), respectively; (**b**) CVs of CeO_2_-phm/cMWCNTs/SPCE in presence of 50 μM H_2_O_2_ recorded at different scan rates.

The electrocatalytic performance of CeO_2_-phm/cMWCNTs/SPCE toward H_2_O_2_ reduction was evaluated by cyclic voltammetry (CV) in 0.1 M PBS (pH 7.0) containing 50 μM H_2_O_2_ at a scan rate of 50 mV·s^−1^. For comparison, CV responses of cMWCNTs/SPCE and CeO_2_-c/cMWCNTs/SPCE were also recorded under identical conditions to serve as control electrodes. As shown in Figure 2a, cMWCNTs/SPCE (curve a), CeO_2_-c/cMWCNTs/SPCE (curve c) and CeO_2_-phm/cMWCNTs/SPCE (curve e) exhibited negligible cathodic current and no discernible reduction peak in PBS without H_2_O_2_. However, compared to curve a, the CV response of cMWCNTs/SPCE in PBS with 50 μM H_2_O_2_ (curve b) also showed no obvious reduction peak, indicating the poor electrochemical response towards H_2_O_2_. As expected, the CeO_2_-c/cMWCNTs/SPCE electrode exhibited a noticeable increase in reduction current only at relatively high overpotentials; however, no distinct cathodic peak corresponding to H_2_O_2_ reduction was observed within the scanned potential window of 0 to –0.8 V. In contrast, the CeO_2_-phm/cMWCNTs/SPCE displayed a pronounced cathodic peak centered at −0.6 V, indicating efficient electrocatalytic reduction of H_2_O_2_. Notably, the peak reduction current at −0.6 V for CeO_2_-phm/cMWCNTs/SPCE was approximately 4.3 times higher than that observed for CeO_2_-c/cMWCNTs/SPCE, underscoring the superior catalytic performance of the porous hollow structure.

The enhanced current response and positively shifted reduction peak potential observed at CeO_2_-phm/cMWCNTs/SPCE can be ascribed to the unique structural characteristics of CeO_2_-phm. To gain insight into the electrochemical reduction mechanism of H_2_O_2_, the influence of scan rate on the CeO_2_-phm/cMWCNTs/SPCE was investigated in the presence of 50 μM H_2_O_2_ (Figure 2b). As the scan rate increased from 10 to 60 mV·s^−1^, a noticeable enhancement in peak current was observed, accompanied by a cathodic peak shift toward more negative potentials. Also, the cathodic peaks are in linear relationship with the square root of the scan rate (Figure S5), which indicates the reduction of H_2_O_2_ at CeO_2_-phm is under diffusion control electrochemical process [37,38]. The excellent electrocatalytic activity toward H_2_O_2_ reduction is mainly attributed to the hollow mesoporous structure and abundant oxygen vacancies. Based on these excellent performances, we can expect that the CeO_2_-phm/cMWCNTs/SPCE-based H_2_O_2_ biosensor has remarkable sensitivity, strong anti-interference performance and outstanding stability.

### 3.3. Electrochemical Sensing Performance of H_2_O_2_

**Figure 3:**
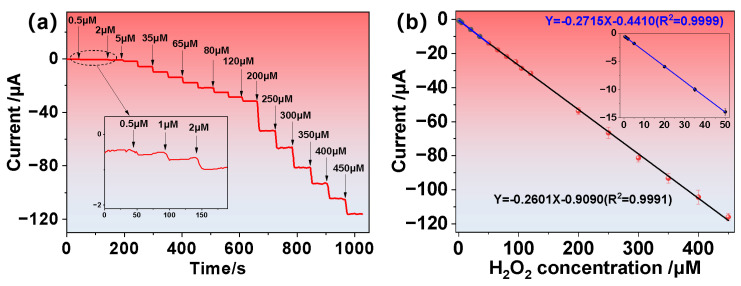
(**a**) Current-time plot for micro CeO_2_-phm/cMWCNTs/SPCE with successive addition of H_2_O_2_ at −0.55 V. (**b**) Corresponding calibration plot between current response and H_2_O_2_ concentration, based on the average of three independent measurements, with error bars representing the standard deviation (mean ± SD).

To optimize the electrochemical performance of the biosensor, the influence of the applied potential on the amperometric response of the CeO_2_-phm/cMWCNTs/SPCE-based H_2_O_2_ biosensor was investigated, revealing that the highest current response was observed at an applied potential of −0.55 V (Figure S6). Consequently, −0.55 V was selected as the optimal operating potential for subsequent electrochemical measurements. The analytical performance of the fabricated biosensor was assessed by constructing a calibration curve. As illustrated in Figure 3a, the amperometric i–t responses were recorded upon successive additions of H_2_O_2_ at a relatively low working potential of −0.55 V (vs. Ag/AgCl). The corresponding calibration plot, depicted in Figure 3b, reveals a well-defined linear relationship between the current response and H_2_O_2_ concentration. The sensor exhibited two distinct linear ranges: 0.5–50 μM and 50–450 μM, with respective sensitivities of 2161.6 μA·mM^−1^·cm^−2^ and 2070.9 μA·mM^−1^·cm^−2^. The presence of two linear ranges is mainly attributed to the transition of the sensing process from a kinetic-controlled regime to a diffusion-controlled regime [39,40], and 50 μM is exactly the critical point. The detection limit was calculated to be 0.017 μM, using a signal-to-noise ratio criterion of 3 (S/N = 3). These performance metrics are either superior to or on par with those reported for other electrochemical H_2_O_2_ biosensors (Table 1), underscoring the excellent sensitivity and wide detection range of the CeO_2_-phm/cMWCNTs/SPCE-based biosensor.

**Figure 4:**
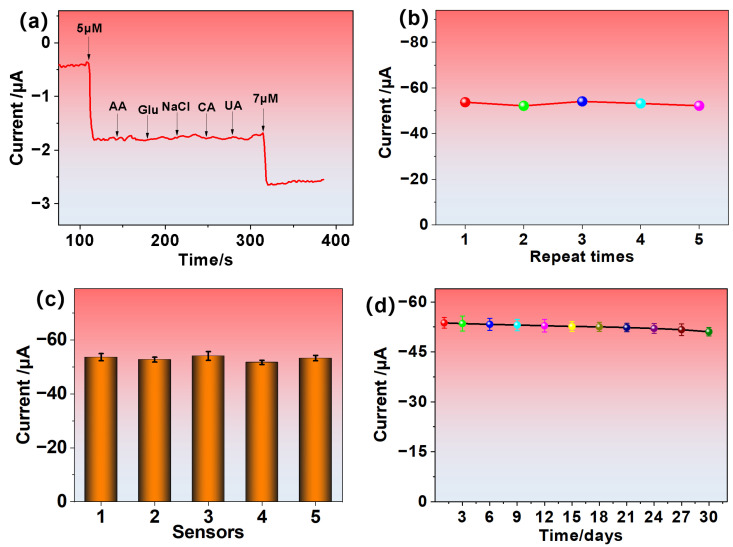
(**a**) Amperometric response of CeO_2_-phm in presence of interference species in 5 μM H_2_O_2_. Supporting electrolyte: 0.1 M PBS (pH 7.0); (**b**) five electrochemical experiments on the same electrode; (**c**) electrochemical tests of five different electrodes under the same conditions; (**d**) electrochemical stability test in 30 days.

To further evaluate the sensing capabilities of the CeO_2_-phm/cMWCNTs/SPCE-based biosensor, its selectivity toward H_2_O_2_ was examined in the presence of common electroactive interferents. As illustrated in Figure 4a, a series of potentially interfering species—such as ascorbic acid (AA), citric acid (CA), uric acid (UA), glucose (Glu), and sodium chloride (NaCl)—were added to a 5 μM H_2_O_2_ solution. The current response remained nearly unchanged following the addition of these species, indicating minimal interference. In contrast, the current response exhibited a distinct stepwise enhancement upon successive additions of H_2_O_2_, which remained consistent even in the presence of potential interfering substances. These results confirm that the CeO_2_-phm-modified electrode possesses excellent selectivity for H_2_O_2_ detection, even in complex environments containing potentially interfering analytes. The excellent anti-interference performance of the CeO_2_-phm/cMWCNTs/SPCE-based biosensor is mainly attributed to the abundant oxygen vacancies of CeO_2_-phm, the hollow mesoporous structure that promotes selective H_2_O_2_ diffusion, the optimized low detection potential, and the synergistic electron transfer with cMWCNTs.

The amperometric responses of CeO_2_-phm/cMWCNTs/SPCE and CeO_2_-c/cMWCNTs/SPCE were evaluated through successive additions of 5 μM H_2_O_2_ in 0.1 M PBS (Figure S7). The results clearly indicate that the CeO_2_-phm-based electrode exhibited a markedly enhanced sensing performance compared to its CeO_2_-c counterpart. This enhancement is primarily attributed to the distinctive porous hollow architecture of CeO_2_-phm, which offers an enlarged electroactive surface area and abundant accessible catalytic sites. These structural advantages promote more efficient electron transfer and enhance the overall electrocatalytic activity toward H_2_O_2_ detection.

The performance consistency of the CeO_2_-phm/cMWCNTs/SPCE sensor was evaluated in terms of repeatability, reproducibility and stability. For repeatability, five successive measurements were carried out using the same electrode, yielding relative standard deviation (RSD) values below 1.68% (Figure 4b). Reproducibility was assessed using five independently fabricated electrodes, with RSD values remaining under 1.73% (Figure 4c). These findings confirm the reliable and consistent performance of the sensor across repeated and independent tests. Additionally, the long-term stability of the sensor was examined by conducting H_2_O_2_ detection every three days over a 30-day period. On day 30, the current response retained 95.04% of its initial value (Figure 4d), indicating strong temporal stability. These results demonstrate that the CeO_2_-phm/cMWCNTs/SPCE biosensor possesses excellent repeatability, reproducibility and stability.

### 3.4. Real Sample Analysis

To evaluate the real-world applicability of the proposed biosensor, hydrogen peroxide was quantified in various complex matrices—including groundwater, commercial drinking water, milk and fetal bovine serum—via the standard addition method [66]. Known concentrations of H_2_O_2_ were spiked into each sample, and the recovery rates were calculated accordingly, as summarized in Table 2. The recoveries ranged from 100.5% to 102.6%, demonstrating the sensor’s high accuracy in complex sample matrices. These results confirm that the CeO_2_-phm/cMWCNTs/SPCE-based biosensor not only performs well under controlled laboratory conditions but also retains its sensitivity and reliability in real-world environments. This highlights its strong potential for use in environmental monitoring and biomedical diagnostics involving H_2_O_2_ detection.

## 4. Conclusions

In summary, in this report, a porous ceria hollow microsphere (CeO_2_-phm) with a uniform pore size (~3.4 nm) and a high specific surface area (168.6 m^2^/g) was synthesized via a facile solvothermal method. These unique structural characteristics, along with abundant oxygen vacancies, significantly enhanced electrocatalytic activity toward H_2_O_2_ reduction. When integrated into a flexible screen-printed electrode functionalized with cMWCNTs, the resulting CeO_2_-phm/cMWCNTs/SPCE biosensor exhibited excellent electrochemical sensing performance, including a wide linear range (0.5–450 μM), an ultralow detection limit (0.017 μM), and a high sensitivity (up to 2070.9 and 2161.6 μA·mM^−1^·cm^−2^). Furthermore, the sensor demonstrated excellent selectivity against common interferents, repeatability and reproducibility (RSD < 1.73%), and long-term stability (retaining over 95% of its signal after 30 days). Importantly, the biosensor was successfully applied in the quantification of H_2_O_2_ in complex real-world samples such as groundwater, milk, commercial drinking water, and fetal bovine serum, with excellent recovery rates (100.5–102.6%). These results collectively validate the CeO_2_-phm/cMWCNTs/SPCE biosensor as a promising platform for real-time, cost-effective, and high-accuracy monitoring of H_2_O_2_ in biomedical diagnostics, environmental surveillance, and food safety applications.

## Figures and Tables

**Table 1:** Comparison of CeO2-phm/cMWCNTs/SPCE-based biosensor with other sensors reported in the literature.

Sensor Material	Operating Potential (V, vs. Ag/AgCl))	Linear Range	Sensitivity	Detection Limit	Reference
CeO_2_-phm/cMWCNTs	−0.55	0.5–50 μM; 50–450 μM	2161.6 μA·mM^−1^·cm^−2^; 2070.9 μA·mM^−1^·cm^−2^	0.017 μM	This work
CeO_2_/Pt/C	−0.4	0.01–30 mM	185.4 ± 6.5 μA mM^−1^ cm^−2^	2 μM	[41]
CeO_2_/rGO xerogel	−0.3	60.7 nM–3.0 μM	1.978 × 10^−1^ μA mM^−1^	30.40 nM	[42]
CeO_2_	−0.3	91.88 μM–2.0 mM	2.9346 × 10^−5^ μA mM^−1^	31.29 μM	[42]
Co/CeO_2_	-	3.33–100 μM; 100–1166 μM; 1166–5000 μM	-	3.33 μM	[43]
CeO_2_/C nanowires	-	0.5–100 μM	-	0.42 μM	[44]
porphyrin functionalized CeO_2_	-	10–100 μM	-	5.29 μM	[45]
porphyrin functionalized CeO_2_ nanorods	-	10–100 μM	-	6.1 μM	[46]
Co_3_O_4_ nanoparticles inside CeO_2_ nanotubes	-	2–80 μM	-	1.2 μM	[47]
La_2_ZnO_4_	-	3.0–85.0 μM	25,000 μA mM^−1^ cm^−2^	0.04 μM	[48]
Phthalocyanine pendented polyaniline	0.015	0.2–52 μM	2317.5 μA mM^−1^ cm^−2^	0.15 μM	[49]
AuNPs-NH_2_/Cu-MOF	−0.15	5–850 μM	1710 μA mM^−1^ cm^−2^	1.2 μM	[50]
Au/ZnO	0.05	1 μM–3.0 mM	1336.7 μA mM^−1^ cm^−2^	0.1 μM	[48]
Fe_3_C@C/Fe-N-C	-	1–6000 μM	1225 μA mM^−1^ cm^−2^	0.26 μM	[51]
Bi_2_S_3_/g-C_3_N_4_	+0.26	0.5–950 μM	1011 μA mM^−1^ cm^−2^	78 nM	[52]
Ag/ZIF-8	−0.6	20 μM–5 mM; 5.5–10 mM	398.47 and 145.21 μA mM^−1^ cm^−2^	6.2 μM	[53]
Prussian blue-polypyrrole composite	−0.1	0–3.5 mM	377.43 μA mM^−1^ cm^−2^	-	[54]
Ag/2D Zn-MOFs	−0.55	5.0 μM–70 mM	358.7 μA mM^−1^ cm^−2^	1.67 μM	[55]
Co-NC/CNF	−0.5	10–5000 μM	300 μA mM^−1^ cm^−2^	10 μM	[56]
Ni_3_Mo_3_N/NC MSs	−0.60	5 μM–40 mM	120.3 μA mM^−1^ cm^−2^	1 μM	[57]
NiMn-LDH/GO	−0.45	20–5860 μM	96.82 μA mM^−1^ cm^−2^	4.4 μM	[58]
Graphene-MWCNT	−0.4	20–2000 μM	32.91 μA mM^−1^ cm^−2^	9.4 μM	[59]
Cu nanoparticles/ERGO	−0.2	0.01–1 mM	20 μA mM^−1^ cm^−2^	1.87 × 10^−9^ M	[60]
CNC-rGO	−0.2	20–160 μM	0.333 μA mM^−1^ cm^−2^	5.28 μM	[61]
Co_3_O_4_ nanowalls	+0.8	0–1.4 mM	100.3 μA mM^−1^	2.8 μM	[62]
Co_3_O_4_ nanowalls	−0.2	0–5.35 mM	4.844 μA mM^−1^	10 μM	[62]
Fe SAs/Co CNs	-	1–400 μM	-	0.36 μM	[63]
Fe–HCl–NH_2_-UiO-66	-	3.125–100 μM	-	1.0 μM	[64]
MIL-47(V)-OH	-	4.38–43.97 μM; 50.33–2240 μM	-	5.84 μM	[65]

**Table 2:** Recovery for the detection of H_2_O_2_ in groundwater, commercial drinking water, milk and fetal bovine serum samples.

Sample	Added (μM)	Relative Standard Deviation (%, n = 3)	Measured (μM)	Recovery (%)
Groundwater	5.0	2.46	5.13	102.6%
Commercial drinking water	2.0	1.34	2.01	100.5%
Milk	1.0	0.96	1.01	101.0%
Fetal Bovine Serum	10	1.02	10.13	101.3%

## Data Availability

The datasets generated during the current study are available from the first author on reasonable request.

## Supplementary Materials

The following supporting information can be downloaded at: https://www.mdpi.com/article/10.3390/bios15100664/s1, Figure S1: FT-IR spectrum of CeO2-phm; Figure S2: XPS analysis of CeO2-phm; Figure S3: (a) EDS layer image, and element mapping of the CeO2-phm representing as (b) Ce, (c) O, (d) N, and (e) C; (f) The EDS representation of the CeO2-phm and insert image showing element composition of the CeO2-phm. Figure S4: The DLS analysis of CeO2-phm; Figure S5: Linear relationship between square roots of canning speed and peak current; Figure S6: Current responses of CeO2-phm/cMWCNTs/SPCEs towards 200 μM H2O2 at different applied operating potential; Figure S7: Amperometric response of continuously addition 5 μM H2O2 for 4 times of CeO2-phm/cMWCNTs/SPCEs (curve a) and CeO2-c /cMWCNTs/SPCEs (curve b).

## Abbreviations

The following abbreviations are used in this manuscript:

CeO_2_-phm	porous ceria hollow microspheres
CeO_2_-c	commercial CeO_2_ nanospheres with solid cores
SPCE	screen-printed carbon electrode
ROS	reactive oxygen species
CeO_2_	cerium dioxide
cMWCNTs	carboxylated multi-walled carbon nanotubes
Ce(NO_3_)_3_·6H_2_O	Cerium nitrate hexahydrate
EG	ethylene glycol
NHS	N-hydroxysuccinimide
EDC	1-ethyl-3-(3-dimethylaminopropyl)carbodiimide hydrochloride
AA	ascorbic acid
Glu	glucose
CA	citric acid
UA	uric acid
NaCl	sodium chloride
PBS	Phosphate-buffered saline
CeO_2_-c	commercial CeO_2_ nanospheres
DLS	dynamic light scattering
PDI	polydispersity index
FBS	Fetal Bovine Serum
