# Supplementary material for: A Flexible Electrochemical Sensor Based on Porous Ceria Hollow Microspheres Nanozyme for Sensitive Detection of H2O2
Source: Biosensors (Basel). 2025 Oct 2;15(10):664. doi: 10.3390/bios15100664 (PMC12563342; doi:10.3390/bios15100664)
Supplement: Supplementary file 1 [file biosensors-15-00664-s001.zip › biosensors-3826953-supplementary.pdf]

Supporting Information

A flexible electrochemical sensor based on porous ceria hollow microspheres nanozyme for sensitive detection of H_2O_2

Jie Huang [1, 2], Xuanda He [1], Shuang Zou [1], Keying Ling [1], Hongying Zhu [1], Qijia Jiang [1], Yuxuan Zhang [3], Zijian Feng [1], Penghui Wang[4], Xiaofei Duan[1], Haiyang Liao [1], Zheng Yuan [5, *], Yiwu Liu [1], Jinghua Tan [1, *]

[1] School of Packaging Engineering, Hunan University of Technology, Zhuzhou, 412007, PR China; huangjie3@alumni.sjtu.edu.cn (J. H.), m24085600021@stu.hut.edu.cn (X. H.), 19374285193@163.com (S. Z.), 13768127226@163.com (K. L.), 15823262606@163.com (H. Z.), 17872584038@163.com (Q. J.), fzj6900@163.com (Z. F.), dxf0923@163.com (X. D.), haiyangliao1990@163.com (H. L.), tjh@hut.edu.cn (J. T.), liuyiwu@hut.edu.cn (Y. L.)

[2] School of Biomedical Engineering, Guangzhou Medical University, Guangzhou, 511436, PR China

[3] Hunan Provincial Key Laboratory of Environmental Catalysis & Waste Recycling, College of Materials and Chemical Engineering, Hunan Institute of Engineering, Xiangtan, 411104, PR China; yuxuanzhang1990@163.com (Y. Z.)

[4] School of Chemistry and Chemical Engineering, State Key Laboratory of Metal Matrix Composites, Shanghai Jiao Tong University, Shanghai 200240, PR China; phwang0622@sjtu.edu.cn (P. W.)

[5] Institute of Chinese Materia Medica, China Academy of Chinese Medical Sciences, Beijing 100022, PR China; zyuan@icmm.ac.cn (Z, Y)

* Correspondence: zyuan@icmm.ac.cn (Z, Y), tjh@hut.edu.cn (J. T.)

Table of Contents:

Figure S1. 3

Figure S2. 3

Figure S3. 4

Figure S4. 4

Figure S5. 5

Figure S6. 5

Figure S7. 6

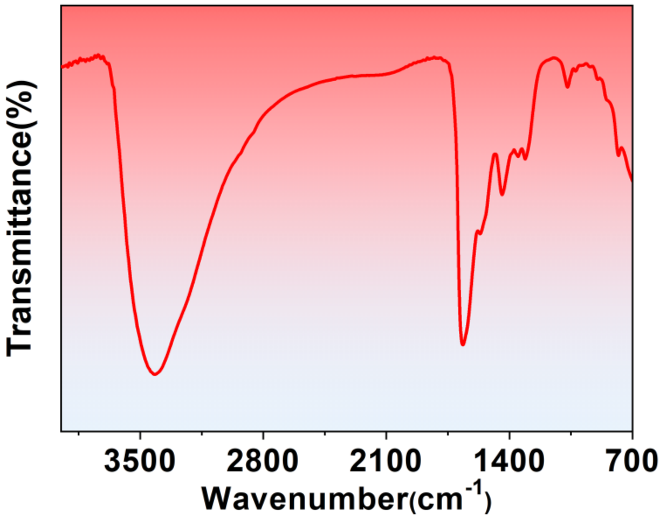

Figure S1 FT-IR spectrum of CeO_2-phm

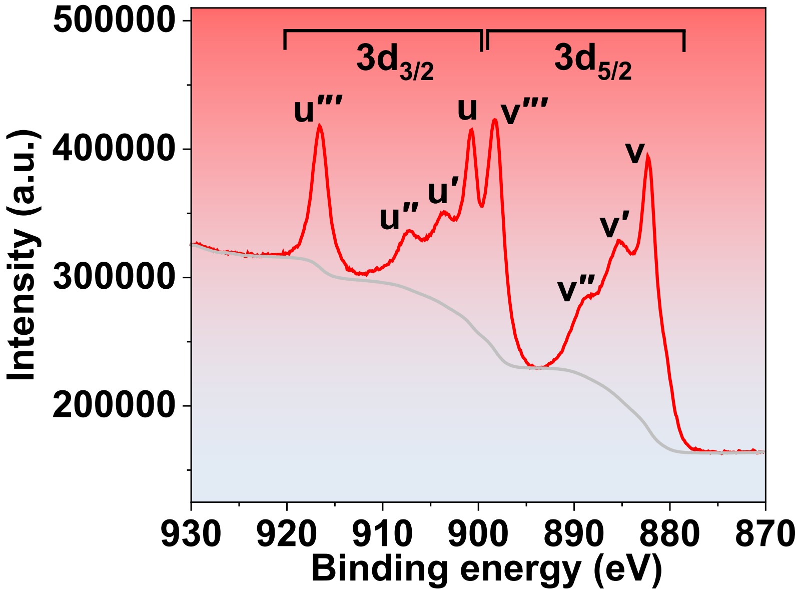

Figure S2. XPS analysis of CeO_2-phm.

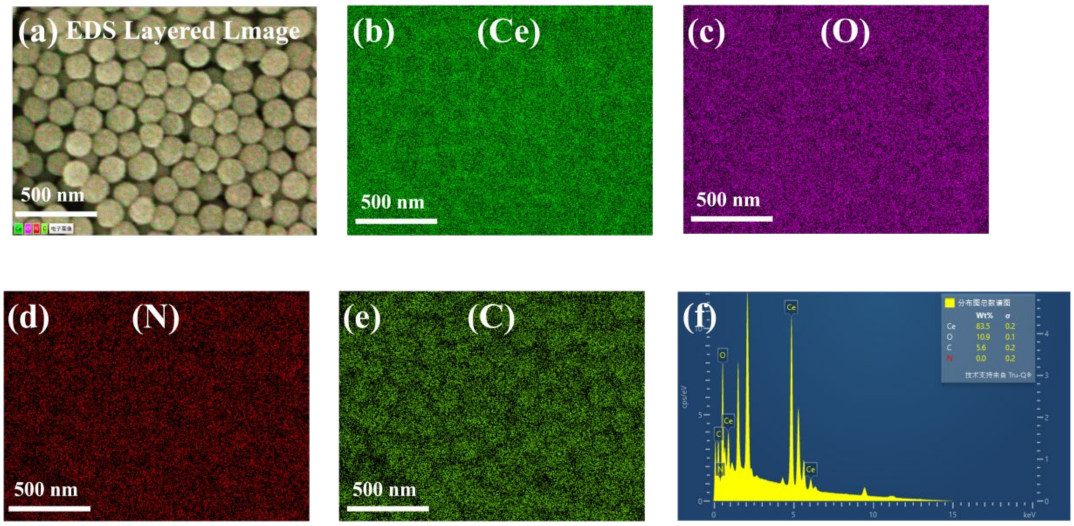

Figure S3. (a) EDS layer image, and element mapping of the CeO_2-phm representing as (b) Ce, (c) O, (d) N, and (e) C; (f) The EDS representation of the CeO_2-phm and insert image showing element composition of the CeO_2-phm.

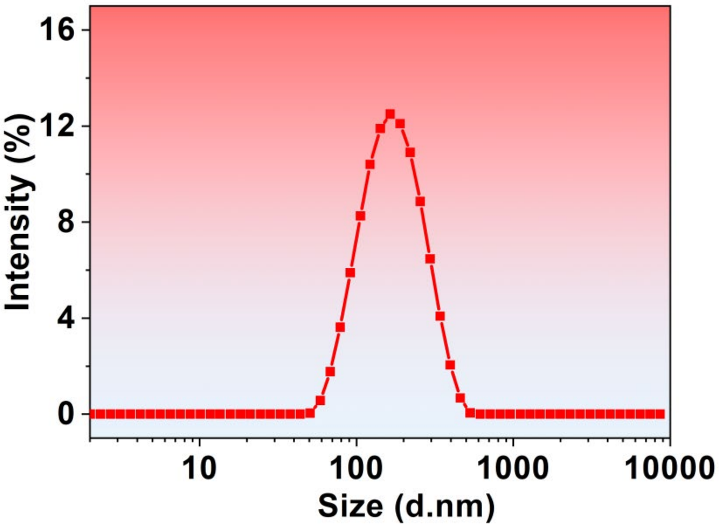

Figure S4. The DLS analysis of CeO_2-phm.

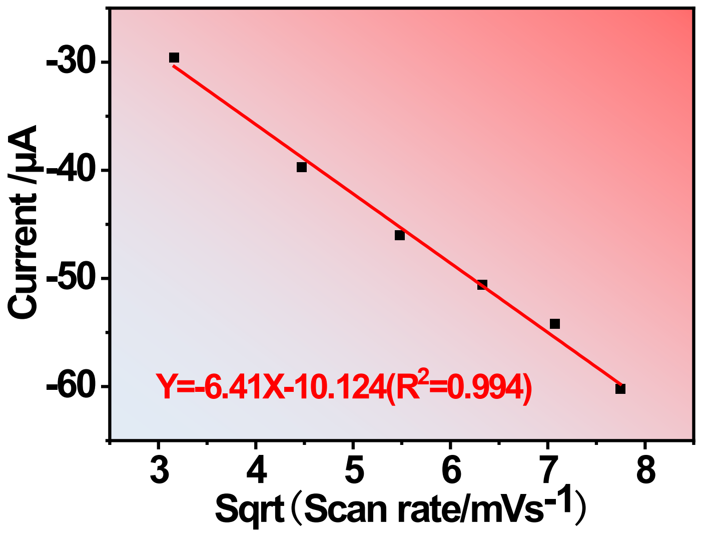

Figure S5. Linear relationship between square roots of canning speed and peak current

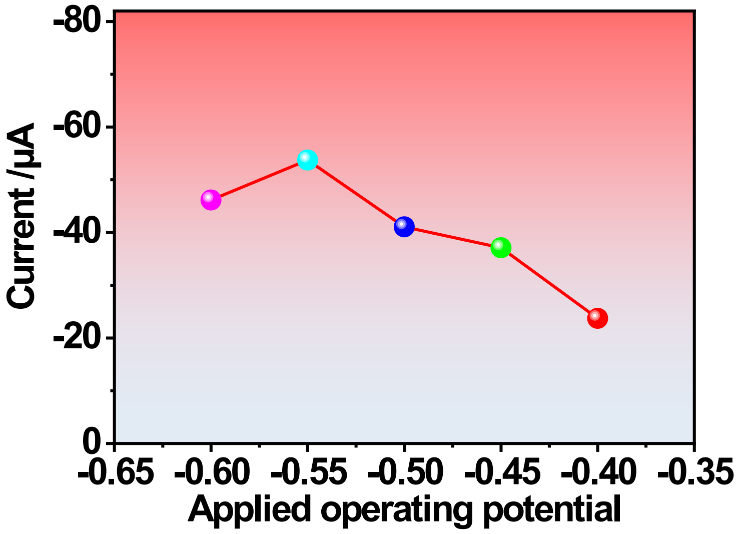

Figure S6. Current responses of CeO_2-phm/cMWCNTs/SPCEs towards 200 μM H_2O_2 at different applied operating potential.

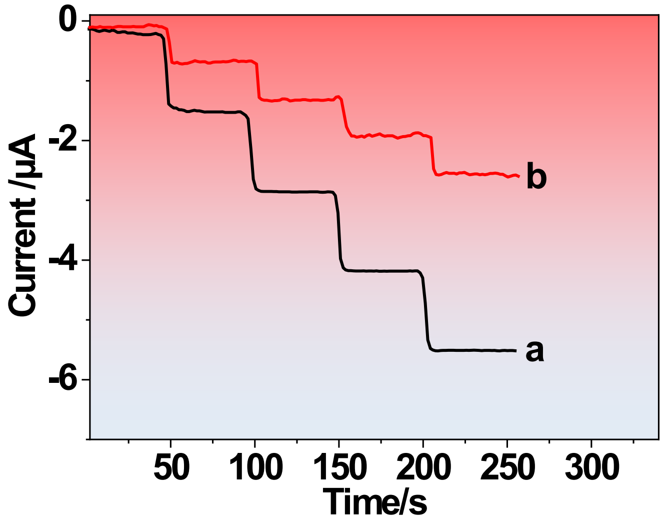

Figure S7. Amperometric response of continuously addition 5 μM H_2O_2 for 4 times of CeO_2-phm/cMWCNTs/SPCEs (curve a) and CeO_2-c /cMWCNTs/SPCEs (curve b)
